# Undergraduate nursing students’ knowledge and experience in infusion therapy and peripheral vascular acces

**DOI:** 10.1590/0034-7167-2022-0219

**Published:** 2023-08-07

**Authors:** Jaciara Tiago Antunes Alvarenga, Adriana Cristina Nicolussi, Aline Maria Pereira Cruz Ramos, Lucas Fernando Antunes Gomes, Damiana Aparecida Trindade Monteiro, Silmara Elaine Malaguti Toffano

**Affiliations:** I Universidade Federal do Triângulo Mineiro. Uberaba, Minas Gerais, Brazil; III Universidade Federal do Pará. Belém, Pará, Brazil

**Keywords:** Nursing, Vascular Access Devices, Knowledge, Education, Nursing, Education, Nursing, Diploma Programs., Enfermería, Dispositivos de Acceso Vascular, Conocimiento, Educación en Enfermería, Programas de Graduación en Enfermería., Enfermagem, Dispositivos de Acesso Vascular, Conhecimento, Educação em Enfermagem, Programas de Graduação em Enfermagem.

## Abstract

**Objectives:**

to analyze the knowledge and experience of undergraduate nursing students regarding infusion therapy and peripheral vascular access.

**Methods:**

descriptive, cross-sectional, analytical study with 123 undergraduate nursing students who answered a semi-structured instrument.

**Results:**

the majority were women, with a median age of 51 years old; 87% considered the teaching received insufficient to perform in clinical practice. The mean overall knowledge score was 78.1 (SD± 8.97). The themes catheter flushing and lock (38.1%), catheter selection (34.2%), infusion equipment (30.9%) and insertion site (30.9%) presented a significant number of errors.

**Conclusions:**

practical classes and execution of procedures in health services were predictors for a better knowledge about infusion therapy and vascular access by undergraduate nursing students.

## RESUMO

**Objetivos:**

analisar o conhecimento e a experiência de graduandos de enfermagem relacionados à terapia infusional e aos acessos vasculares periféricos.

**Métodos:**

pesquisa descritiva, transversal, analítica, com 123 estudantes do curso de graduação em enfermagem que responderam a um instrumento semiestruturado.

**Resultados:**

a maioria, mulheres, com mediana de 51 anos; 87% consideraram o ensino recebido insuficiente para atuar na prática clínica. A média de pontos geral de conhecimento foi de 78,1 (DP± 8,97). Os temas *flushing* e *lock* do cateter (38,1%), seleção do cateter (34,2%), equipamentos para infusão (30,9%) e sítio de inserção (30,9%) apresentaram número significativo de erros.

**Conclusões:**

aulas práticas e realização de procedimentos nos serviços de saúde foram preditores para um melhor conhecimento sobre terapia infusional e acesso vascular pelos estudantes do curso de graduação em enfermagem.

## RESUMEN

**Objetivos:**

analizar el conocimiento y experiencia de estudiantes avanzados de enfermería sobre terapia infusional y accesos vasculares periféricos.

**Métodos:**

investigación descriptiva, transversal, analítica; con 123 estudiantes del último curso de enfermería, que respondieron a un instrumento semiestructurado.

**Resultados:**

mayoría de mujeres, mediana de 51 años; el 87% considera insuficiente la enseñanza recibida para desempeñarse en la práctica clínica. El promedio general de puntos de conocimiento fue del 78% (DE± 8,97). Los temas *flushing* y *lock* del catéter (38,1%), selección del catéter (34,2%), equipos de infusión (30,9%) y punto de inserción (30,9) mostraron un número significativo de errores.

**Conclusiones:**

clases prácticas y realización de procedimientos en servicios de salud fueron el camino a un mayor conocimiento en terapia infusional y acceso vascular para los estudiantes del último curso de enfermería.

## INTRODUCTION

Vascular access (VA) is one of the main treatment modalities used in health care, for numerous purposes, such as administration of solutions, anesthesia and invasive monitoring^(1-3)^.

The theoretical basis and the development of skills regarding VA and infusion therapy (IT) since graduation^(2-6)^ play an important role for quality nursing care practice. In this regard, previous results point to the need for evaluation of the evolution of undergraduate nursing students over the years of training^(1-2,5,7)^. Considering that nursing is increasingly evolving in advanced practices in VA, a solid education is important for professional practice^(7)^.

According to the Resolution of Curricular Guidelines for Undergraduate Nursing Courses^(8)^, nurses should have the necessary set of contents, competencies and skills to promote the capacity for autonomous and permanent intellectual and professional development, and curricular conceptions must be monitored and permanently evaluated in order to allow the necessary adjustments for their improvement.

Thus, it is necessary to identify the weaknesses that undergraduate nursing students have during training, with the development of strategies for monitoring the knowledge and skills acquired about procedures and care with VA and handling of IT, in order to promote the consolidation of learning, updating on the issues and encouraging their constant improvement^(2)^.

## OBJECTIVES

To analyze the knowledge and experience of undergraduate nursing students regarding infusion therapy and peripheral vascular access.

## METHODS

### Ethical aspects

The ethical aspects were respected according to resolution number 466, December 12, 2012, of the National Research Council, and the project was submitted to the Research Ethics Committee for review and approval. All participants accessed and signed an informed consent form to participate in the study.

### Study design, period and setting

This is a descriptive, cross-sectional, analytical study with a quantitative approach. It was conducted between March 1, 2020, and December 15, 2021.

The study site was a Brazilian public higher education institution, whose course curriculum provides contact with the theme since the first period, with topics on hand hygiene, health professional safety, and safe environment. From the third year on, students have access to theoretical content on IT and VA, as well as practical classes in skills laboratories, so they can later exercise nursing care safely in health services. During this period, technical aspects related to the nurses’ specific attributions are also covered. It is important to emphasize that, in the fifth period, the peripheral venipuncture (PVP) technique is taught. All students finish the content at the end of the fourth year and carry out their mandatory supervised internship in the fifth year.

The recommendations of the Strengthening the Reporting of Observational Studies in Epidemiology (STROBE) were followed for the preparation of the manuscript.

### Population, sample, and inclusion and exclusion criteria

The inclusion criteria were being 18 years of age or older, being enrolled in an undergraduate nursing course, having access to the Internet, and having completed the specific discipline involving techniques and procedures related to VA and IT in the fifth period.

The population consisted of 155 students. The sample size calculation considered the coefficient of determination of R^2^=0.13 in a multiple linear regression model with 7 predictors, with a significance level or type I error of α=0.05 and type II error of β=0.2, resulting, therefore, in an aprioristic statistical power of 80%.

After entering the values described above in the Power Analysis and Sample Size (PASS) application, version number 13, a minimum sample size of n = 104 was obtained. Considering the sampling loss of 20%, the final number of interview attempts was n = 130.

### Study protocol

To conduct the study, a semi-structured instrument was designed with questions about IT and VA. An apparent validation was performed by three experts, who are PhDs and have 4 to 17 years of experience in the subject^(9-10)^, who met the following inclusion criteria: having at least a master’s degree and publications in the field of intravenous therapy. They were selected based on the Lattes Platform data and invited by e-mail. After acceptance, they signed an informed consent form and answered an instrument prepared and sent by Google Forms, due to the COVID-19 pandemic. The final instrument, with 110 questions on VA and IT and 13 questions on demographic and academic variables, was obtained after two rounds of evaluation and agreement on the items and content.

After the authorization of the responsible instances and the Ethics Committee, the researchers had access to the institutional e-mail and telephone number of the students. Once they were clarified of the research objectives and accepted to participate in the study, the participants signed the informed consent form and had access to the data collection instrument by the Google Forms virtual platform.

### Analysis of results and statistics

The data were organized in a database in Excel for Windows software (Microsoft Inc.) and exported to IBM^®^ Statistical Package for the Social Sciences (SPSS) software. Subsequently, they were analyzed using descriptive and inferential statistics. Spearman’s correlation was used to evaluate the grade, according to the year of study, due to the difference in the presentation of numeric variable x numeric (grade/year) and the t-test was used for the remaining variables.

A score of 0 to 110 was considered to evaluate the knowledge of the undergraduate nursing students. Thus, it was determined that the greater the number of correct answers in the totality of questions, the greater the knowledge on the topic. Finally, the influence of demographic variables, experience and knowledge of undergraduate nursing students on the topic was analyzed using multiple linear regression and bivariate analysis.

## RESULTS

The sample was made up of 123 undergraduate nursing students, with seven refusals of the informed consent form and 25 undergraduates who did not return after two contact attempts. The majority were female (n=115/93.5%), in their fourth year (n=46/37.4%) and not working in the healthcare field (n=112/91.1%). Their median age was 51 years (SD±18.504), with a minimum of 24 and a maximum of 80 years (Table 1).

**Table 1:** Characterization of participants (N=123) according to sex, age group, year of study, and undergraduate health workers, Uberaba, Minas Gerais, Brazil, 2021

Variable	n	%
Sex		
Female	115	93.5
Male	08	6.5
Age group (in years)		
≤ 20	09	7.3
21-25	96	78.0
26-29	07	5.7
30-35	06	4.9
36-40	02	1.6
≥ 40	03	2.4
Course year		
3º	44	35.8
4º	46	37.4
5º	33	26.8
Worked in the healthcare field		
Sim	11	8.9
Não	112	91.1

Among the 125 participants, 11 (8.9%) reported having a professional occupation concomitant with their graduation, eight of them were nursing technicians, with one to 14 years of professional experience (Table 2).

**Table 2:** Frequency analysis of right and wrong answers of undergraduate nursing students from a public federal university (N=123), by theme of the evidence-based instrument, Uberaba, Minas Gerais, Brazil, 2021

Theme	Answer	
	Right (n/%)	Wrong (n/%)
About infusion therapy practice	112 (91.0)	11 (9.0)
Catheter material	86 (69.9)	37 (30.1)
Infusion system and accessories	95 (77.2)	28 (22.8)
Infusion equipment	85 (69.1)	38 (30.9)
Vascular visualization technologies	100 (81.3)	23 (18.7)
Hypodermoclysis	86 (69.9)	37 (30.1)
Intraosseous infusion	89 (72.3)	34 (27.7)
Parenteral nutrition	103 (88.6)	20 (11.4)
Infection prevention	89 (72.3)	34 (27.7)
Site of insertion	85 (69.1)	38 (30.9)
Catheter selection	81 (65.8)	42 (34.2)
Preparation of the insertion site	98 (79.6)	25 (20.4)
Catheter stabilization	93 (75.6)	30 (24.4)
Coverage	93 (75.6)	30 (24.4)
Daily care/evaluation	95 (77.2)	28 (22.8)
Equipment cleaning and disinfection	90 (73.1)	33 (26.9)
Catheter flushing and lock	84 (68.2)	39 (31.8)
Transfusion therapy	92 (74.7)	31 (25.3)
Drugs used in infusion therapy	107 (86.9)	16 (13.1)
Catheter removal	100 (81.3)	23 (18.7)
Patient safety	91 (73.9)	32 (26.1)
Waste generated in infusion therapy	101 (82.1)	22 (17.9)

In general, the participants said they had attended theoretical classes on the theme (n=111/ 90.2%), practical activities in health services (n=103/ 83.7%), classes (n=64/ 52.0%) and complementary courses (n=105/ 85.4%). The majority considered the teaching received insufficient to work in clinical practice (n=107/87.0%).

Regarding practical activities, most reported that they had contact with the theme in health services (n=103/83.7%), such as in Basic Health Units (n=48/ 25.2%), hospitals (n=09/8.7%) or both (n=09/8.7%). Regarding the practice of PVP in a patient, a little more than half of the participants had the opportunity to perform a puncture (n=63/51.2%) and, among those, a minority was successful in the procedure (n=47/38.2%).

The overall mean score for VA and IT knowledge was 78.1 points (SD ± 8.975) and median of 80 with a minimum of 51 and a maximum of 98 points.

Undergraduate nursing students who took practical classes in health services related to the topic of IT and VA had higher scores than those who did not (80.5 SD± 7.909 and 74.8 SD± 9.370, respectively), showing a statistically significant association between the groups (t(121) = 0.067; p< 0.000). Spearman’s correlation showed that there is a positive and weak correlation between grade and years of study in the undergraduate nursing course (*p* = 0.047; *p*= 0.606), according to Table 3.

**Table 3:** Association of variables with adequate and inadequate knowledge according to the t-test and Spearman’s correlation of undergraduate nursing students from a public federal university (N=123), Uberaba, Minas Gerais, Brazil, 2021

Variable	n	Mean overall score	SD (±)	CI (min-max)	*p*
Sex					
Female	115	78.8	8.961	-0.399^*^ (-7.83360-5.20534)	0.691
Male	08	79.3	9.709		
Professional occupation					
Yes	11	79.4	11.518	0.196^*^ (-4.19488-7.06825)	0.614
No	112	78.0	8.740		
Theoretical classes					
Yes	111	78.5	9.115	0.137^*^ (-1.52130-9.23301)	0.158
No	12	74.6	6.919		
Complementary classes					
Yes	64	76.6	8.556	0.344^*^ (-6.27770-0.06478)	0.055
No	59	79.7	9.208		
Practical classes					
Yes	71	80.5	7.909	0.067^*^ (2.59218-8.77564)	0.000
No	52	74.8	9.370		
Courses					
Yes	105	77.5	8.831	0.712^*^ (-8.48811-0.5000)	0.081
No	18	81.5	9.300		
Course year					
≤ 3rd	44	-	-	0.047^**^	0.606
4th	46	-	-		
≥ 5th	36	-	-		

* T-Test;

** Spearman’s correlation; plevel of significance: p <005.

According to the logistic regression with seven predictors (sex, occupation, theoretical class, practical class, complementary classes, year of the nursing course, and attendance of courses on the subject), all of them rejected the hypothesis of a relationship with adequate knowledge in IT and VA.

## DISCUSSION

Most participants were female, corroborating the final report of a study on the profile of nursing in Brazil^(10)^ and the results of other studies^(11-13)^.

It was expected that most students in the undergraduate nursing course had already taken a theoretical class on the subject, as pointed out in the study, considering that the subject involves techniques and procedures, in the fifth period, and further develops the student’s knowledge on the topic of VA and IT.

Concomitant with the theoretical class on the topic, it was also expected that most participants had performed practical activities in health services related to IT and VA. This is due to the fact that Brazilian universities sign partnerships for practice programs, such as internships for the training of undergraduate nursing students.

In this study, it was prevalent that most students of the undergraduate course in nursing took complementary classes and courses on VA and IT, since the university under study promotes teaching, research and extension, ratifying the large participation in extracurricular activities, which provide significant moments of learning, being expected that they develop fundamentals and critical thinking on the subject more deeply^(13)^.

Most participants had performed a PVP in a patient; however, it is worth noting the large number of undergraduate nursing students, even at different stages of training, who had never attempted to puncture a PVP, a fact also noted in other studies^(14-15)^.

For undergraduate nursing students to be efficient in the performance of the procedure, they must build skills throughout their studies^(14,16)^. The construction of skills requires the combination of theoretical, practical, and attitudinal knowledge, simultaneously^(17)^. Provided with prior experience, undergraduate nursing students’ fear of the procedure is reverted into knowledge and subsequently into safety in future experiences^(18)^.

Different teaching methods make it possible to contextualize the subject dynamically and actively, in order to relate theory and practice with the expansion of learning content such as PVP^(16-19)^. These alternatives make it possible to perform the procedure in a controlled situation before performing it on the patient, providing early experience in a safe manner, further development of technical skills and decreased anxiety when later performing it in real scenarios^(16-19)^.

Regarding the theme “catheter material”, there was a large number of errors in the answers regarding the type of material. Considering that the catheter material is directly linked to the occurrence of complications such as phlebitis and infiltration, due to its specificity, it is expected that undergraduate nursing students had knowledge about its composition, the manufacturer’s technical recommendations, and general guidelines^(20-21)^.

With regard to knowledge about technologies for vascular visualization, the participants pointed to a lack of knowledge about vascular ultrasound (VUS), equipment that allows visualization of veins for insertion of various types of catheters, including in central veins^(19-21)^. Despite advances in VUS for VA, this is still a recent technology for Brazilian nurses, which requires training so they can acquire sufficient skills and competencies for successful puncture using this technology^(22)^.

Regarding infection prevention, the participants’ lack of knowledge about the item that discusses the importance of not performing routine replacements in arterial accesses, PVP and central venous catheters, according to the literature, was noteworthy^(18-23)^. It is still essential that clinical evaluation be the criterion for performing PVP replacements^(20-21)^.

Regarding the issue of insertion site, there were different answers regarding the recommendations of the region for the first installation of a peripheral venous catheter. PVP is one of the main attributions of the nursing team; however, doubts are still observed by professionals, a fact that interferes negatively in health care and especially in patient safety, lacking educational initiatives since graduation for the reduction in rates of bloodstream infection and phlebitis^(20-21,24)^.

In the catheter selection theme, one of the items addressed the installation of the PVP not being considered a private act of nurses. This presented a considerable number of errors, and it is important to reinforce that the prescription of intravenous therapy is a medical act, and the choice of catheter is a multidisciplinary decision, since nurses evaluate the conditions of the venous network and contribute significantly to this decision, based on the need for the type of solution, infusion time, and other clinical variables^(20-21)^.

As for catheter stabilization, the participants pointed out some relevant aspects that should not be performed, such as reintroducing the catheter when there is external migration, contrary to what is recommended, where the catheter should not, under any circumstances, be reintroduced when it is externally migrated^(20-22)^.

Regarding the theme that deals with the VA cover, the answers of the participants also pointed out different recommendations as to the time of replacement of the semi-permeable transparent film, which, according to the literature, should be within 7 days^(20-21)^. Regarding the experience with the procedure of changing the sterile semi-permeable transparent film, the majority answered that they had never performed the procedure, a fact that may justify, in part, the lack of familiarity and knowledge specifically on the issue.

Regarding patient safety, specifically in the item about ‘always remove from the unit’s common stock drugs that are considered “potentially dangerous” or of high risk^(19-20)^’, the recommendation is that, besides the removal of these medications from the care units, the ampoules found should always be identified with warning labels, alerting that the medication can be fatal^(23)^.

Only carrying out practical classes in health services related to IT and VA (p < 0.000) was indicated by the t-test as a predictor of better knowledge about the topic of study by the participants. In view of the above, it is evident the importance of developing skills so that, throughout the course, the difficulties presented during this process, such as living in an unknown and complex environment, insecurity, difficulty in working in groups, differences in the reception of the professional team, lack of inputs and poor professor monitoring are overcome with the development of methods aimed at solving these gaps, with a view to direct learning outcomes^(24-25)^.

Focus should be on reflective teaching, which stimulates and helps undergraduate nursing students to build their skills and competencies, for the construction of their professional identity, achievement of autonomy, leadership, conflict resolution, decision-making, management and management in nursing^(25)^. The proposition of effective strategies, such as increasing the number of practical classes, internship time, possible attempt to perform more procedures, patient contact time, and more active teaching methodologies, like realistic simulation, aims at a better and more complete visualization of professional nursing practice for students^(24-25)^.

In view of the above, further research is needed, in different institutions, to expand discussions on the subject and improve the quality of education, in view of the needs of Brazilians, and rethinking the reality of the curricular structure of health courses, especially in nursing.

### Study limitations

The fact that students from different periods were included, depending on the opportunities in the practical classes, made it possible to have different experiences with the research theme, which may have contributed to the positive or negative results. The use of an instrument with many questions may also influence the answers; however, the participants were informed in advance about the number of questions and expected response time. Other relevant aspects were: only students who had access to the Internet participated in the study; and the non-conduction of a pilot test.

It is worth mentioning that, in the years 2020 and 2021, disciplines had to be adapted, due to the pandemic, when students of the undergraduate course in nursing received part of the theoretical classes remotely (online) and there was also a 50% reduction in the number of face-to-face practical classes, which limited the possibility of practical experiences for them.

### Contributions to the field

For higher educational institutions, it is essential to identify the deficits in the knowledge of undergraduate nursing students regarding IT and VA, which influence the training process, to enable the further development of methodologies with the necessary adjustments to achieve the transition from being a graduate student to being a professional. Thus, by strengthening research, offering subsidies for improving teaching, reflecting directly on future nursing professionals, it will be possible to promote the prevention and early identification of complications in VA, improving health care for the population.

## CONCLUSIONS

Most of the participants had scores above average and those who had practical classes or performed procedures in health services scored higher. This was also a predictor for better knowledge about IT and VA by undergraduate nursing students.

## Data Availability

https://doi.org/10.48331/scielodata.Z1NSA4

## References

[1] Ahlin C, Klang-Sodervist B, Johansson E, Bjorkholm M, Lofmark A. (2017). Assessing nursing students' knowledge and skills in performing venipuncture and inserting peripheral venous catheters. Nurse Educ Pract.

[2] Melo GSM, Tibúrcio MP, Freitas CCS, Vasconselos QLDAQ, Costa IKF, Torres GV. (2017). Semiologia e semiotécnica da enfermagem: avaliação dos conhecimentos de graduandos sobre procedimentos. Rev Bras Enferm.

[3] Oliveira ASS, Costa PJS, Graveto JMGN, Costa FJG, Osório NIA, Cosme ASTC. (2019). Nurses’ peripheral intravenous catheter-related practices: a descriptive study. Referência.

[4] Simonetti V, Comparcinia D, Miniscalcoc D, Tirabassid R, Di Giovannie IP, CicolinifI G. (2019). Assessing nursing students' knowledge of evidence-based guidelines on the management of peripheral venous catheters: a multicentre cross-sectional study. Nurse Educ Pract.

[5] Frota NM, Galindo NNM, Barros LM, Pereira FGF, Melo GAA, Caetano JA. (2018). Hypermedia on peripheral venipuncture: effectiveness in teaching nursing students. Rev Bras Enferm.

[6] Vasconcelos JO, Faria JGM, Cury-Borim B, Truzzi IGC, Jericó MC. (2019). Conhecimento dos estudantes de enfermagem no estágio de auditoria de assistência em um hospital de ensino. Unifunec Ciênc Saúde Biol.

[7] Massey D, Craswell A, Ray-Barruel G, Ullman A, Marsh N, Wallis M. (2020). Undergraduate nursing students' perceptions of the current content and pedagogical approaches used in PIVC education: a qualitative, descriptive study. Nurse Educ Today.

[8] Ministério da Educação (BR) (2001). Resolução CNE/CES 3/2001.

[9] Gilbert GE, Prion S. (2016). Making Sense of Methods and Measurement: Lawshe’s Content Validity Index. Clin Simul Nurs.

[10] Taylor E. (2020). We Agree, Don't We? the Delphi Method for Health Environments Research. HERD.

[11] Machado MH (2017). Pesquisa Perfil da Enfermagem no Brasil: relatório final.

[12] Colichi RMB, Gómez-Urrutiall V, Nunes HRC, Lima SAM. (2020). Perfil e intenção empreendedora de estudantes de enfermagem: comparativo entre Brasil e Chile. Rev Bras Enferm.

[13] Aguiar KLA, Vieira MA, Domenico EBL. (2021). Avaliação de egressos de cursos de graduação em enfermagem: estudo brasileiro multicêntrico. Rev Enferm USP.

[14] Larsen EM, Byrnes J, Marsh N, Rickard CM. (2021). Patient-reported outcome and experience measures for peripheral venous catheters: a scoping review protocol. Braz J Nurs.

[15] Mota SP, Nascimento JS, Azevedo SPBM, Freitas CCS, Feijão AR, Melo GSM. (2019). Punção venosa periférica: análise dos registros de acadêmicos de enfermagem. Rev Enferm UFSM.

[16] Canever BP, Sanes MS, Oliveira SN, Magalhães ALP, Costa DG. (2021). Metodologias ativas no cateterismo periférico venoso: desenvolvimento de habilidades com simulador de baixo custo. Esc Anna Nery.

[17] Santana BS, Paiva AAM, Magro MCS. (2020). Skills acquisition for safe drug administration through realistic simulation: integrative review. Rev Bras Enferm.

[18] Pedrada LDSA, Brum AKR, Moraes EB, Suzart RGDC, Pinto AZLBC. (2021). Use of realistic simulation in the safety of the surgical team against coronavirus: experience report. Res, Soc Dev.

[19] Ministério da Saúde (BR) (2017). Medidas para prevenir infecções relacionadas aos cuidados de saúde.

[20] Carrara D, Polastrini RTV, Infusion Nurses Society Brasil (INS BRASIL) (2018). Diretrizes Práticas para Terapia Infusional.

[21] Gorski LA, Infusion Nurses Society (INS) (2021). Infusion Therapy Standards of Practice.

[22] Lima HC, Lenhani BE, Batista J, Heimbecher CT. (2021). Experiência de estudantes de enfermagem na técnica de punção venosa periférica com e sem o uso de transiluminador cutâneo portátil. Res, Soc Dev.

[23] Santos AV, Pantoja AC, Dantas AS, Garcia JV, Cruz ER, Conceição CM (2021). Recomendações nacionais a cateteres periféricos: análise do conhecimento da equipe de enfermagem em um hospital universitário na Amazônia Brasileira. Enferm Foco.

[24] Instituto para práticas seguras no uso de medicamentos Brasil (ISMP Brasil) (2019). Boletim ISMP Brasil: medicamentos potencialmente perigosos de uso hospitalar. Lista atualizada.

[25] Dantas FM, Souza HF, Silva TA, Matos PB, Silva JEB, Albuquerque FHS. (2020). Relevância do estágio curricular em Hospital Universitário sob a perspectiva de estudantes de enfermagem do interior do Amazonas. Braz J Health Rev.

