# ERRATUM

**DOI:** 10.1590/0034-7167.20237606e06

**Published:** 2023-12-04

In the article “Undergraduate nursing students’ knowledge and experience in infusion therapy and peripheral vascular acces”, with DOI number: https://doi.org/10.1590/0034-7167-2022-0219, published in Revista Brasileira de Enfermagem, 2023;76(3): e20220219, authored:

Where it read:

**Adriana Cristina Nicolussi^II^**

**ORCID: 0000-0001-5600-7533**

*^II^Universidade Federal do Pará. Belém, Pará, Brasil*.

It reads:

**Adriana Cristina Nicolussi^I^**

**ORCID: 0000-0001-5600-7533**

*^I^Universidade Federal do Triângulo Mineiro. Uberaba, Minas Gerais, Brasil*.

